# Foliar Application of Silicon and Sulfur Modifies Grain Mineral Composition of Spring Oats ( *Avena sativa* L.) Under Contrasting Seasonal Drought Conditions

**DOI:** 10.3390/plants15020316

**Published:** 2026-01-21

**Authors:** Bekir Bytyqi, Fanni Zsuzsa Forgács, Anteneh Agezew Melash, István Csaba Virág, József Csajbók, Ebenezer Ayew Appiah, Erika Tünde Kutasy

**Affiliations:** 1 Institute of Crop Production, Breeding and Plant Technology, Faculty of Agricultural and Food Sciences and Environmental Management, University of Debrecen, Böszörményi út 138, H-4032 Debrecen, Hungary; bekir.bytyqi@uni-pr.edu (B.B.); forfazsu@agr.unideb.hu (F.Z.F.); antenehagezew@dku.edu.et (A.A.M.); virag.istvan.csaba@agr.unideb.hu (I.C.V.); csj@agr.unideb.hu (J.C.);; 2 Department of Food Technology and Biotechnology, Faculty of Agriculture and Veterinary, University of Prishtina, Str. “Tahir Zajmi”, No. 34, 10000 Prishtina, Kosovo; 3 Department of Horticulture, College of Agriculture and Environmental Science, Debark University, North Gondar, Debark P.O. Box 90, Ethiopia

**Keywords:** *Avena sativa* L., cultivar response, genotype × environment, drought, silicon nutrition, sulfur nutrition, macroelements, microelements, mineral accumulation

## Abstract

This study evaluated the effects of foliar silicon (Si) and sulfur (S) applications under contrasting climatic conditions on macro- and micronutrient accumulation in oat grain. The three-year field experiment (2022–2024) was conducted in Debrecen, Hungary, using a randomized complete block design (RCBD)with three replications. Grain samples were analyzed for macroelements (K, P, S, Mg, Ca) and micronutrients (Na, Si, Fe, Mn, Cu). Environmental conditions markedly influenced nutrient accumulation. Severe drought promoted the highest concentrations of K, S, and Mg, while mild drought significantly increased the accumulation of P, Ca, Si, Fe, and Cu contents. Moderate drought favored Na accumulation. Foliar S application under relatively favorable water supply significantly enhanced the concentration of all measured elements, with the strongest response observed for Cu (+47.4% compared with the control) and the weakest for Mg (8.5%). In contrast, Si application alone had only limited or negative effects, particularly under severe drought, where it reduced K (6.4%), S (2.4%), and Ca (13%) concentrations, despite increased Si accumulation in the grain. During drought stress, however, the combined Si + S treatment significantly increased the grain macro- and micronutrient concentrations. Among the tested genotypes, ‘Mv Pehely’ exhibited the highest macronutrient accumulation, while ‘GK Kormorán’ and ‘Mv Pehely’ showed superior micronutrient accumulation. ‘GK Pillangó’ and ‘Mv Szellő’ showed consistently lower nutrient contents. These results highlight the importance of genotype × environment × nutrient management strategies for improving nutrient composition in oat grain.

## 1. Introduction

Cereals play a central role in human nutrition, providing more than fifty percent of the daily caloric intake in human diets and serving as important sources of essential mineral nutrients and bioactive compounds. Among cereal crops, oat (*Avena sativa* L.) has gained increasing attention due to its high nutritional value and health-promoting properties [1]. Oat grain is rich in carbohydrates, proteins, dietary fiber, lipids, and a wide range of macro- and micronutrients, including phosphorus (P), magnesium (Mg), manganese (Mn), zinc (Zn), copper (Cu), and iron (Fe) along with smaller amounts of calcium (Ca) and potassium (K) [2,3,4,5,6]. These mineral elements play key physiological roles in human health, particularly in glucose regulation [7], cardiovascular functions [8], bone metabolism [9,10,11,12], protein synthesis [13,14], and enzymatic processes, thereby contributing to the growing interest in oat-based foods and biofortified cereal products.

Climatic change poses a major challenge for sustainable cereal production through rising temperatures and altered water availability. The occurrence of extreme environmental conditions increases the risk of crop failures (yield and quality) in oat production. Drought stress not only reduces crop yield but also substantially alters nutrient uptake, translocation, and accumulation in cereal grains [15]. Previous studies have demonstrated that mineral composition is strongly influenced by both stress intensity and genotype, resulting in considerable variability in grain elemental profiles across environments [16,17]. Consequently, improving grain nutritional quality under increasingly frequent drought conditions has become a key objective in cereal research.

Agronomic management practices, particularly fertilization strategies, play a critical role in mitigating the adverse effects of abiotic stress and improving grain mineral composition [18]. Foliar fertilization is considered an efficient approach for supplying nutrients directly to plants, especially under conditions where soil nutrient availability or root uptake is limited by drought stress [19]. Targeted foliar applications can therefore serve as an efficient management tool to enhance nutrient accumulation and stabilize grain quality across variable environmental conditions.

Among foliar-applied nutrients, silicon and sulfur have received increasing attention in cereal production. Silicon is widely reported to enhance plant tolerance to abiotic stresses, including drought, by improving water-use efficiency, strengthening cell walls, and modulating nutrient uptake [20,21,22,23,24,25]. Several studies, mainly in wheat, have shown that foliar Si application under drought conditions can increase the accumulation of macroelements (K, Ca, P, Mg, and S) and micronutrients (Na, Si, Fe, Cu, Mn) in grain [22,23,24,25]. However, accumulating evidence suggests that Si-induced responses are highly conditional, depending on genotype, stress severity, nutrient interactions, and environmental context, and may not always result in positive outcomes. In contrast, sulfur is an essential nutrient involved in protein synthesis, enzymatic activity, and redox regulation, and its adequate supply has been consistently associated with in improved grain productivity and quality in cereals [26,27]. Additionally, adequate sulfur application enhances nutrient uptake in cereals under diverse environmental conditions [26].

Despite the recognized importance of Si and S in cereal nutrition, information on their foliar application in oats, particularly under contrasting drought conditions and across different genotypes, remains limited. Moreover, studies simultaneously addressing the interactions among genotype, environment, and nutrient management (G × E × M) in determining oat grain mineral composition are scarce. Understanding these interactions is essential for developing effective fertilization strategies and selecting genotypes with stable nutritional performance under climate-induced stress conditions.

Therefore, the aim of this study was to evaluate the effects of foliar silicon and sulfur applications on the accumulation of macroelements (K, S, P, Mg, Ca) and microelements (Na, Fe, Cu, Mn, Si) in oat grain under contrasting environmental conditions across multiple growing seasons. We hypothesized that (i) the effects of foliar sulfur and silicon application on oat grain mineral composition are strongly dependent on environmental conditions and genotype and that (ii) combined silicon and sulfur application may modify nutrient accumulation differently under contrasting drought intensities, resulting in significant genotype × environment × management interactions.

## 2. Results

### 2.1. Assessment of Drought Severity in the Experimental Years Using the Pálfai Drought Index (PaDI)

The modified Pálfai Drought Index (PaDI) is an excellent indicator for characterizing drought severity using a single numerical value across different agricultural years. The PaDI is computed using hydrological years (October–September) and integrates temperature data with weighted monthly precipitation totals. In terms of drought, not only the precipitation conditions of the given year are decisive, but also those of the preceding period, which is why the precipitation amounts of the preceding years (36 months) are also taken into account as a correction factor. This drought index’s main advantage lies in its simple calculation, which relies on readily available meteorological data (monthly average temperature, monthly sum of precipitation). The resulting value provides a reliable assessment of drought intensity and its potential impact on crop yields. (Table 1).

Based on the PaDI value, the year 2023 is classified as experiencing mild drought (PaDI = 4.3 °C 100 mm^−1^), while 2024 is characterized as moderate drought (PaDI = 6.5 °C 100 mm^−1^) The 2022 growing season is considered the most unfavorable, with a value of 11.6 °C 100 mm^−1^, indicating a serious drought (Table 1 and Table 2).

### 2.2. Effects of Silicon and Sulfur Foliar Treatments, Genotype, and Environment on Macroelement Concentrations in Spring Oat Grain

#### 2.2.1. Effects of Silicon and Sulfur Foliar Treatments and Environment on Macroelement Concentrations in Spring Oat Grain

**Figure 1:**
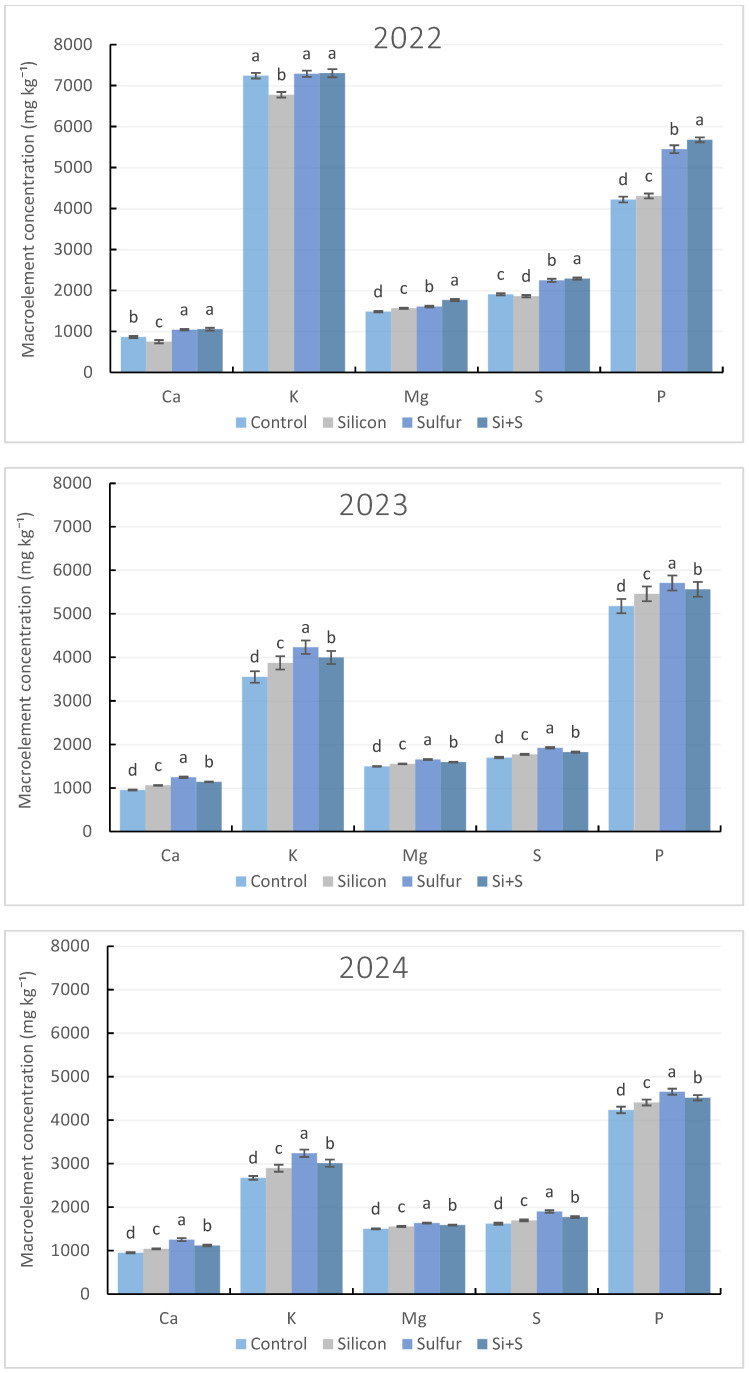
Macroelement content in spring oat whole grains depending on foliar treatments with Si, S, and Si + S during three growing seasons (2022–2024). Columns represent annual mean values of varieties (8) and repetitions (3) ± standard error (n = 24). Different letters indicate statistically significant differences among treatments within the same macroelement content, based on LSD test at *p* ≤ 0.05.

Macroelements such as potassium (K), calcium (Ca), magnesium (Mg), sulfur (S), and phosphorus (P) were analyzed in oat grain samples. Potassium and phosphorus were present at markedly higher concentrations than S, Mg, and Ca. Macroelements varied significantly across years and foliar treatments (*p* < 0.05; Figure 1), indicating strong seasonal and treatment-dependent effects on nutrient accumulation.

Foliar treatments resulted in distinct macroelement response patterns depending on water availability. In seasons with relatively adequate water availability (2023 and 2024), foliar sulfur application significantly increased the concentrations of all analyzed macroelements. Under serious drought stress (2022), however, the combined Si + S treatment resulted in the highest accumulation of K, P, S, Mg, and Ca, while Si alone had no consistent positive effect.

Grain potassium concentration was primarily determined by water availability, showing a strong and significant negative correlation with total precipitation during the growing period (*r* = −0.663), and, consequently, a strong significant positive relationship with the Pálfai Drought Index (*r* = 0.696). Potassium levels were markedly elevated in the drought year (2022) compared with the more favorable seasons. In severe drought, a significantly lower grain K concentration was detected under the silicon treatment, whereas the control, sulfur, and combined Si + S treatments exerted statistically indistinguishable potassium concentrations. In mild and moderate drought all applied treatments resulted in significantly higher potassium concentrations compared with the control. Across all three years, sulfur application consistently produced the most favorable effect on potassium accumulation, irrespective of water availability, while the combined Si + S treatment demonstrated a similarly beneficial impact (Figure 1).

In contrast, grain phosphorus concentration exhibited a positive relationship with water availability; however, sulfur-containing treatments consistently enhanced grain P content irrespective of seasonal conditions. The effect of each treatment differed significantly across all years, with the lowest phosphorus values consistently recorded in the control treatment.

Similar to K, a negative relationship (*r* = −0.475) was observed between water availability during the growing season and grain sulfur content. Sulfur-based treatments had a positive effect on the sulfur concentration in the grain. S concentrations were significantly higher under the Si + S foliar application in 2022 season, whereas sole S foliar application resulted in increased S accumulation for 2023 and 2024 seasons.

Magnesium and calcium concentrations were significantly affected by foliar treatments (*r* = 0.578 and *r* = 0.497). The combined Si + S foliar application was particularly effective under drought stress, whereas sulfur alone resulted in higher Mg and Ca accumulation under more favorable moisture conditions. Across all seasons, control plots consistently showed the lowest Mg and Ca contents.

Overall, silicon alone did not substantially improve macroelement accumulation in spring oat grain. Sulfur-based foliar treatments proved most effective, with combined Si + S performing best under drought stress and sole application of sulfur under adequate water supply.

#### 2.2.2. Effects of Genotype and Environment on Macroelement Concentrations in Spring Oat Grain

**Figure 2:**
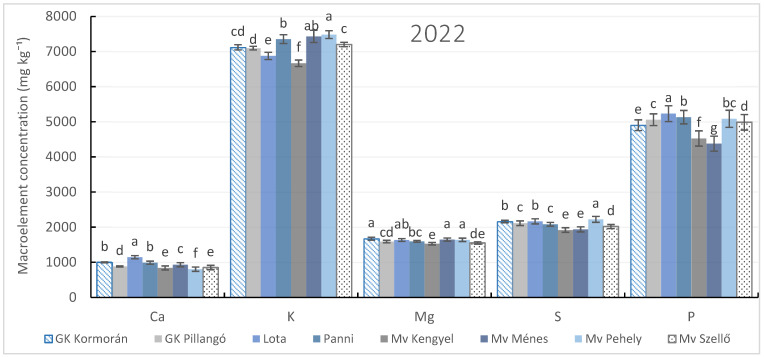

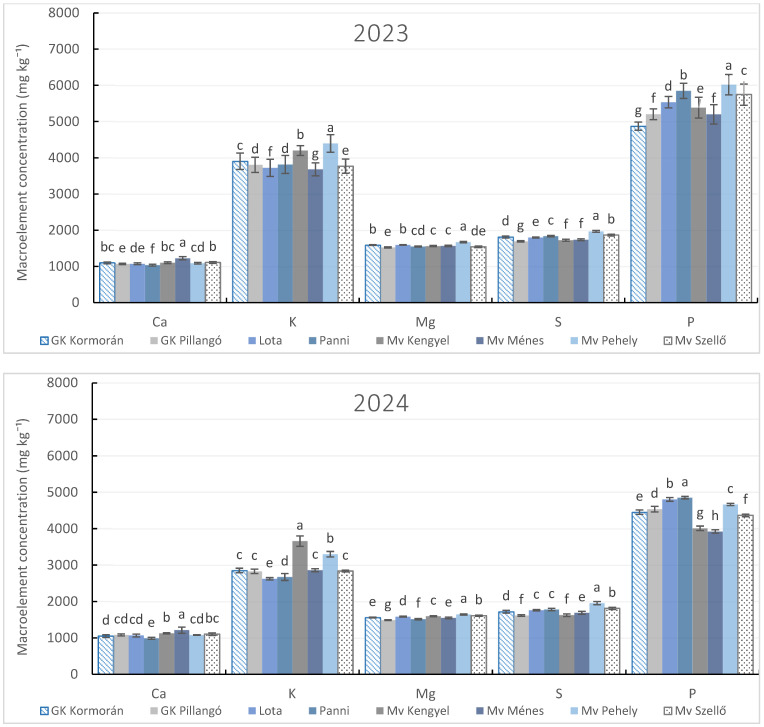
Macroelement content in spring oat whole grains depending on varietal divergences during three growing seasons (2022–2024). Columns represent annual mean values of treatments (4) and repetitions (3) ± standard error (n = 12). Different letters indicate statistically significant differences among varieties within the same macroelement content, based on LSD test at *p* ≤ 0.05.

The results presented in Figure 2 demonstrate that macroelement concentrations varied significantly among varieties and seasons (*p* < 0.05), indicating pronounced genotype- and year-dependent effects on nutrient accumulation.

Potassium concentration showed substantial variability across both varieties and years, with generally higher values observed in severe drought (2022) and the lowest levels in 2024 across all genotypes. Certain varieties, particularly ‘Mv Pehely’, consistently exhibited higher K concentrations across seasons, highlighting the combined influence of genotype and environmental conditions (Figure 2).

Phosphorus accumulation also differed markedly among genotypes and seasons. Averaged across years, the 2023 season was favorable for all varieties. Among the genotypes, ‘Mv Pehely’, ‘Lota’, and ‘Panni’ showed the highest phosphorus accumulation, indicating genotype-specific responses to seasonal variability.

A stable varietal pattern was observed for sulfur and magnesium accumulation, with the ‘Mv Pehely’ variety consistently exhibiting the highest grain S and Mg concentrations across all examined seasons Additionally, the ‘GK Kormorán’ variety had the highest Mg content in 2022, indicating its potential as a competitive alternative under specific environmental conditions.

In contrast, calcium accumulation showed a stronger variety × season interaction, with different genotypes exhibiting peak Ca concentrations depending on the year. Ca concentration was highest in the ‘Lota’ variety in 2022), whereas ‘Mv Ménes’ exhibited greater Ca accumulation in 2023 and 2024.

These findings highlight the strong influence of variety × environmental interactions on nutrient dynamics in spring oat and underscore the necessity of season-specific nutrient management strategies to optimize macroelement accumulation across different spring oat genotypes.

### 2.3. Effects of Silicon and Sulfur Foliar Treatments, Genotype, and Environment on Microelement Concentrations in Spring Oat Grain

#### 2.3.1. Effects of Silicon and Sulfur Foliar Treatments and Environment on Microelement Concentrations in Spring Oat Grain

**Figure 3:**
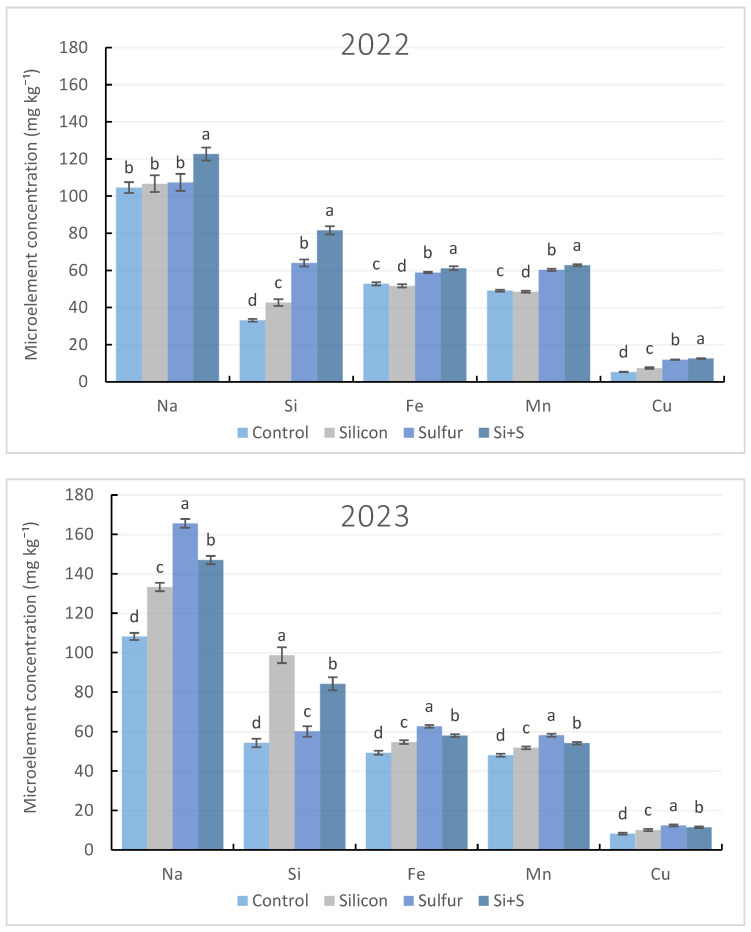

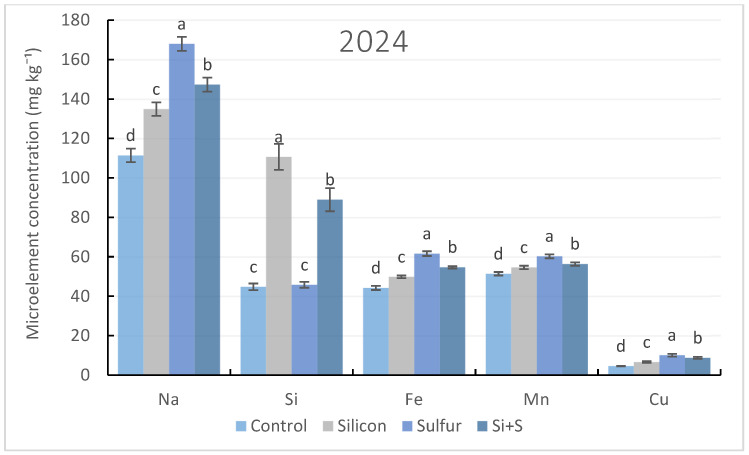
Microelement content in spring oat whole grains depending on foliar treatments with Si, S, and Si + S during three growing seasons (2022–2024). Columns represent annual mean values of varieties (8) and repetitions (3) ± standard error (n = 24). Different letters indicate statistically significant differences among treatments within the same microelement content, based on LSD test at *p* ≤ 0.05.

Microelements, including sodium (Na), silicon (Si), iron (Fe), manganese (Mn), and copper (Cu), were also analyzed in oat grain samples. Figure 3 illustrates the effects of treatments, averaged across genotypes, analyzed separately for each year. All applied treatments resulted in a significant increase (*r* = 0.368–0.596, *p* ≤ 0.01) in micronutrient concentrations. Overall, Na, Si, Fe, and Mn concentrations were present at higher concentrations than Cu.

Microelement concentrations in spring oat grain varied across seasons, with notable trends observed for sodium (Na), silicon (Si), iron (Fe), manganese (Mn), and copper (Cu). Analysis of the data indicates that different seasons and foliar treatments caused statistically significant differences in the accumulation of these microelements. Under severe drought conditions (2022), the combined Si + S treatment produced the highest accumulation of all analyzed micronutrients. In contrast, under more favorable moisture conditions (2023–2024), sulfur application was most effective in increasing Na, Fe, Mn, and Cu concentrations, while Si alone consistently increased only Si content.

Among micronutrients, grain Na concentration showed the strongest relationship with water availability during the growing season. A moderate, positive, and statistically significant correlation was observed between total precipitation (March–July) and grain Na concentration (*r* = 0.440). In contrast, grain iron concentration was only weakly affected by seasonal precipitation (*r* = 0.152). In drought, sole Si application had a slightly negative effect on Fe accumulation, reducing grain Fe content by 2.2% compared with the control (Figure 3).

These results highlight the crucial role of specific foliar treatments and seasonal conditions in influencing microelement uptake. Overall, untreated control plots never reached the maximum microelement concentrations. Foliar Si application alone was effective only in maximizing Si content in 2023 and 2024, whereas the combined Si + S treatment produced significant increases across all studied microelements in 2022. Additionally, the data underscore the importance of sulfur foliar treatment in optimizing microelement content, particularly for Na, Fe, Mn, and Cu, in spring oat grain.

#### 2.3.2. Effects of Genotype and Environment on Microelement Concentrations in Spring Oat Grain

**Figure 4:**
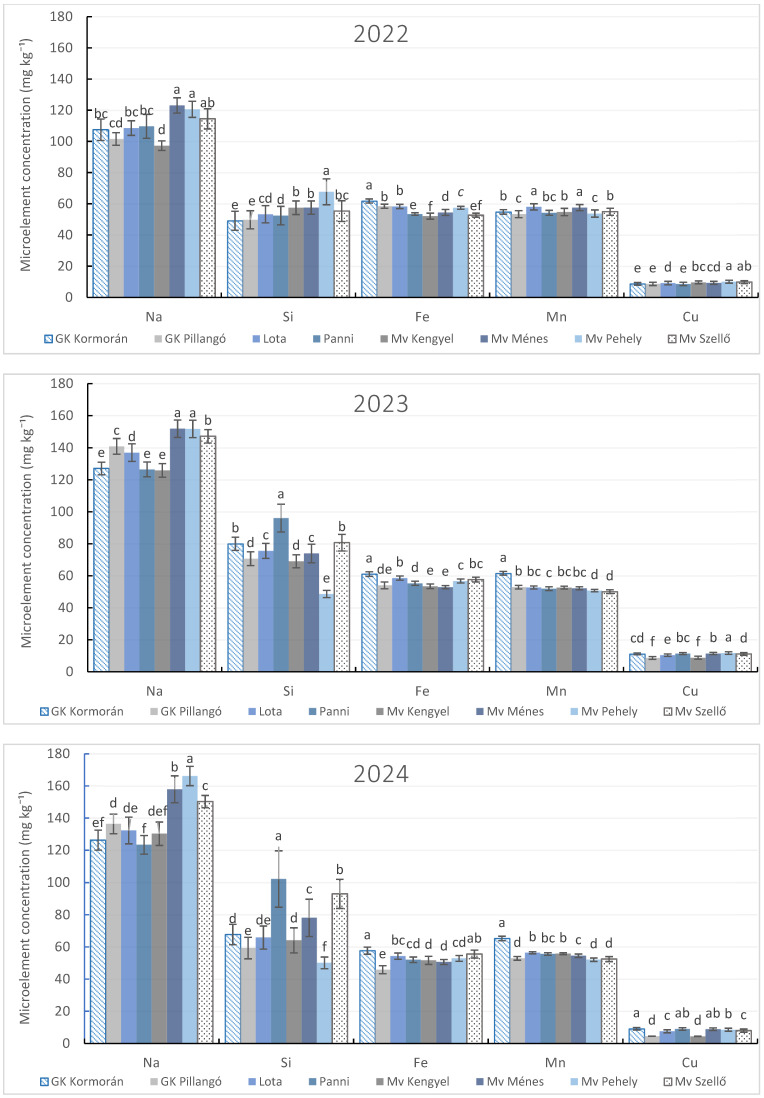
Microelement content in spring oat whole grains depending on varietal divergences during three growing seasons (2022–2024). Columns represent annual mean values of treatments (4) and repetitions (3) ± standard error (n = 12). Different letters indicate statistically significant differences among varieties within the same microelement content, based on LSD test at *p* ≤ 0.05.

Micronutrient concentrations (Na, Si, Fe, Mn, and Cu) in spring oat grain varied significantly among varieties and seasons, indicating pronounced genotype × season interactions (Figure 4).

Sodium accumulation showed considerable varietal and seasonal variability, with certain genotypes (notably ‘Mv Pehely’ and ‘Mv Ménes’) consistently exhibiting significantly higher Na concentrations depending on the year. Silicon content also differed significantly among varieties, with ‘Panni’ showing the highest Si accumulation in favorable seasons, highlighting the importance of variety selection for enhancing Si accumulation in spring oat grain. In severe drought ‘Mv Pehely’ exhibited the highest Si concentration, whereas in 2024 it showed the lowest accumulation across all genotypes.

Iron accumulation was strongly genotype-dependent, with ‘GK Kormorán’ consistently exhibiting the highest Fe concentration across seasons. The lowest Fe concentrations were observed in ‘Mv Kengyel’ in 2022, ‘Mv Ménes’ in 2023, and ‘GK Pillangó’ in 2024. Manganese and copper concentrations showed marked seasonal shifts in varietal ranking, with different genotypes achieving peak accumulation depending on the year.

Overall, these results demonstrate that micronutrient accumulation in spring oat grain is strongly influenced by genetic background and seasonal conditions, underscoring the relevance of genotype-specific responses in optimizing grain nutritional quality.

### 2.4. Macro- and Microelement Concentrations as Affected by the Treatments, Varietal Differences, and Environmental Conditions

Macro- and microelement concentration of whole grain differed significantly among the tested spring oat varieties (*p* < 0.05). The study revealed pronounced variations in both macro- and microelement contents across the varieties. Among all tested genotypes, ‘Mv Pehely’ exhibited the highest mean values for K (5425 mg kg^−1^), P (5710 mg kg^−1^), S (2053 mg kg^−1^), Mg (1662 mg kg^−1^), and Cu (11.1 mg kg^−1^). However, ‘Mv Pehely’ displayed comparatively low Si content, with a mean of 55 mg kg^−1^, whereas the highest Si concentration was observed in ‘Panni’ (81.5 mg kg^−1^). The greatest Ca and Na concentrations were recorded in ‘Mv Ménes’ (1127 and 142 mg kg^−1^, respectively). Regarding microelements, ‘GK Kormorán’ demonstrated exceptionally high Fe (61.4 mg kg^−1^) and Mn (59.3 mg kg^−1^) levels relative to the other varieties. In contrast, ‘GK Pillangó’ exhibited the lowest concentrations of several macroelements and Cu, while the lowest Si and Fe contents were observed in ‘Mv Ménes’ (Table 3). Analysis of variance indicated that all examined macro- and microelements were highly significant (*p* < 0.01).

Among the treatments, sulfur foliar application resulted in the highest concentrations of selected macroelements, including K (5200 mg kg^−1^), P (5623 mg kg^−1^), S (2032 mg kg^−1^), and Ca (1179 mg kg^−1^). In addition, sulfur treatment achieved the highest levels of microelements, namely Na (146 mg kg^−1^), Fe (61.5 mg kg^−1^), Mn (58.9 mg kg^−1^), and Cu (12.3 mg kg^−1^). Combined Si + S foliar application produced the highest accumulation of Mg (1651 mg kg^−1^) and Si (83.3 mg kg^−1^) in spring oat grain. The lowest concentrations of all macro- and microelements were observed in untreated control plots. The interaction between variety and foliar treatment showed the following trend in influencing element accumulation: S > Si + S > Si > Control (Table 4).

The results from the 2022, 2023, and 2024 seasons indicate highly significant effects of climatic conditions on both macro- and microelement concentrations in oat grain, as presented in Table 5. Severe drought conditions had a pronounced impact on the accumulation of macronutrients such as K, S, and Mg. In contrast, mild drought conditions influenced the concentrations of P as well as microelements including Si, Fe, Mn, and Cu in spring oat grains. Moderate drought parameters, however, had a significant positive effect on Na accumulation.

### 2.5. Treatment × Variety × Year Interactions

The highly significant Treatment × Variety × Year interactions, together with the very large effect sizes (partial η^2^ = 0.85–0.88 for Ca, Cu, P, and Si), indicate that genotype-specific responses to treatments were strongly modulated by year-to-year environmental variation. This highlights the dominant role of year-dependent conditions in shaping nutrient accumulation patterns and suggests limited temporal stability of treatment effects. In contrast, Fe concentration exhibited a smaller, though still substantial, three-way interaction effect (partial η^2^ = 0.46), implying a comparatively more stable response across years (Table 6).

### 2.6. Pearson Correlation Analysis Results of Treatments, Sum of Precipitation During the Growing Season, PaDI Values, and Element Content in Spring Oat Whole Grains

The correlations among treatments, sum of precipitation during the growing season (March–July), PaDI values, and element concentrations in whole spring oat grains were analyzed (Table 7). Pearson correlation analysis revealed a significant moderate positive correlation between treatments and grain Ca, Cu, Fe, Mg, Mn, Na, and S content (*r* = 0.437–0.596, *p* ≤ 0.01), as well as a significant but weak positive correlation between treatments and grain P and Si contents (*r* = 0.288–0.368, *p* ≤ 0.01).

Grain K content was not affected by the treatments; however, it showed a highly significant negative correlation (*r* = −0.663, *p* ≤ 0.01) with the total precipitation during the growing season and a highly significant positive correlation (*r* = 0.696, *p* ≤ 0.01) with the calculated Pálfai Drought Index (PaDi) values. Similar but moderate significant correlations were observed for S content with precipitation (*r* = −0.475) and PaDI (*r* = 0.494).

Improved water supply positively affected grain Si, Cu, Na, Ca, and P contents, as indicated by positive correlations with precipitation (*r* = 0.328 to 0.497) and PaDI (*r* = −0.330 to−0.465). Low but significant negative correlations were observed between K and Ca (*r* = −0.281, *p* ≤ 0.01) and between K and Na (*r* = −0.342, *p* ≤ 0.01).

The analysis also revealed strong positive correlations between Ca and Na (*r* = 0.684, *p* ≤ 0.01), P and Cu (*r* = 0.796, *p* ≤ 0.01), S and K (*r* = 0.687, *p* ≤ 0.01), S and Mg (*r* = 0.629, *p* ≤ 0.01), and Fe and Cu (*r* = 0.678, *p* ≤ 0.01) content in whole spring oat grain.

### 2.7. Principal Component Analysis Results of Silicon and Sulfur Treatments on Macro- and Microelement Contents of Spring Oat Grains

**Figure 5:**
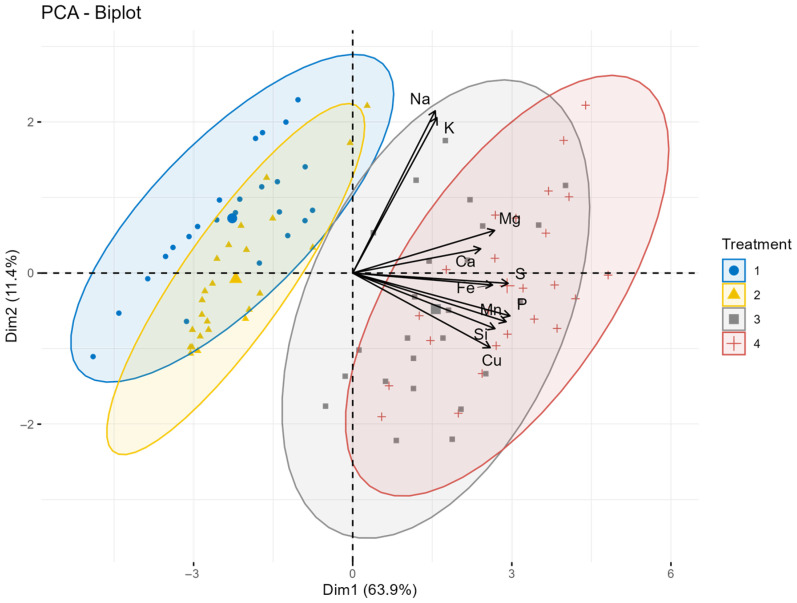
The principal component analysis biplot illustrating the relationship of treatments on macro- and microelement content of oat grain in 2022. The first principal component (Dim1) explains 63.9% of the variance, while the second principal component (Dim2) contributes 11.4%, making a total variability of 75.3%. Treatments: 1 = control, 2 = Si, 3 = S, 4 = Si + S foliar treatments.

The results from the Principal Component Analysis (PCA) investigating the element contents of oats grain in the extremely dry 2022 year can be seen in Figure 5. The biplot indicates that the application of sulfur and combined silicon + sulfur foliar treatments significantly impacts oat grain’s quality. The distinct clustering of the control and silicon treatment groups from the sulfur and silicon + sulfur groups suggests that sulfur and silicon + sulfur treatments have an effect on the plant’s biochemical profile. The first two principal components (Dim1 and Dim2) explain 75.3% of the total variance, with Dim1 accounting for 63.9% and Dim2 for 11.4%. A clear separation is observed along Dim1, the control, and the silicon treatments separate from sulfur and silicon + sulfur application. The PCA biplot clearly shows the differences between the leaf treatment groups based on the macro- and microelement composition of oat grain in 2022. Dim1, which explains 63.9% of the variance, primarily distinguishes the control and Si treatment groups from the S and Si + S treatments, indicating that sulfur-containing treatments had the strongest effect on the elemental profile of the grains. Dim2 (11.4%) shows a further, albeit significantly smaller, difference between treatments.

Elemental loading vectors reveal that K, Mg, Ca, and Fe are strongly associated with the S and Si + S treatments, suggesting enhanced accumulation of these nutrients following sulfur-based foliar applications. In contrast, Cu and Zn show closer alignment with the control and Si-only treatments. Overall, the PCA demonstrates that the combined Si + S treatment produced the most distinct multivariate response, highlighting the interactive role of silicon and sulfur in regulating nutrient composition in oat grain under drought stress conditions.

**Figure 6:**
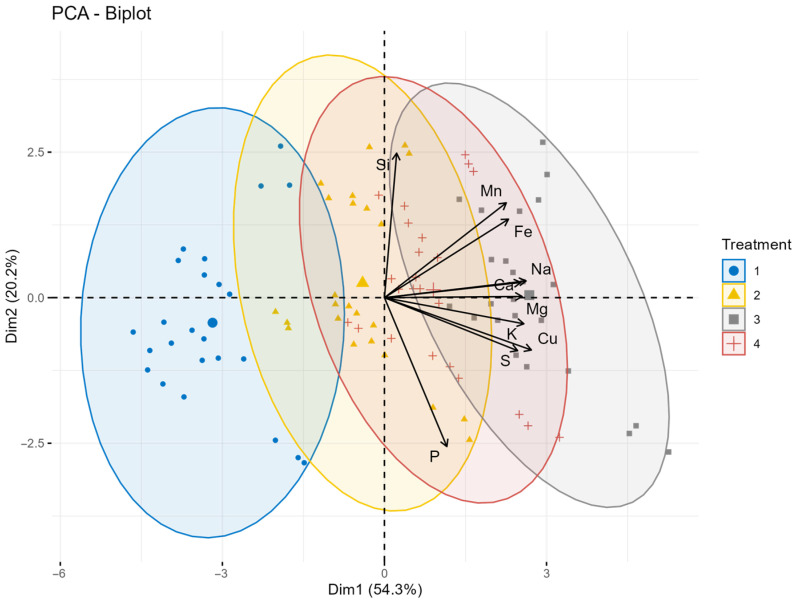
The principal component analysis biplot illustrating the relationship of treatments on macro- and microelement content of oat grain in 2023. The first principal component (Dim1) explains 54.3% of the variance, while the second principal component (Dim2) contributes 20.2%, making a total variability of 74.5%. Treatments: 1 = control, 2 = Si, 3 = S, 4 = Si + S foliar treatments.

The 2023 Principal Component Analysis diagram (Figure 6) shows a partially overlapping separation between leaf treatments based on the macro- and microelement composition of oat grains. Dim1 accounts for 54.3% of the total variance and primarily distinguishes the S and Si + S treatments from the control and Si-only treatments. Closely related to this axis are the elements P, S, Mn, Fe, Mg, Si, and Cu, all of which show high positive loadings (0.81–0.93), indicating that sulfur-containing treatments significantly enhance the accumulation of these elements. Dim2 explains a further 20.2% of the variance and contributes to the separation mainly through K and Na, both of which show strong positive loadings (0.647 and 0.676, respectively). Overall, the 2023 multivariate sample shows that S, and especially the combined Si + S foliar fertilization, resulted in the most pronounced element profiles. Compared to 2022, the contribution of Dim2 increased, reflecting a more heterogeneous treatment response for K and Na. Nevertheless, as in the previous year, sulfur-containing treatments remained the dominant factors in the variation in grain element composition, particularly affecting P, S, Mn, Mg, and Fe concentrations.

**Figure 7:**
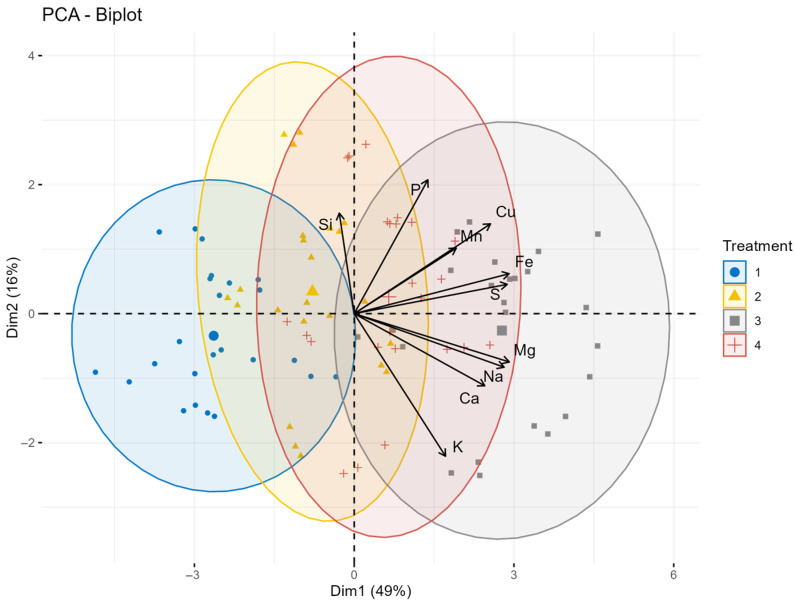
The principal component analysis biplot illustrating the relationship of treatments on macro- and microelement content of oat grain in 2024. The first principal component (Dim1) explains 49.0% of the variance, while the second principal component (Dim2) contributes 16.0%, making a total variability of 65.0%. Treatments: 1 = control, 2 = Si, 3 = S, 4 = Si + S foliar treatments.

The 2024 PCA results indicate a well-defined multi-element separation between canopy treatments (Figure 7). Dim1 explains most of the variability (49%) and is closely related to the elements Fe, Mg, S, and Na (loadings 0.846–0.875). Ca, Cu, and Mn also contribute positively to the first group, albeit with slightly lower loadings. In contrast, Si shows a near-zero and slightly negative loading on this axis, suggesting that silicon content did not follow the main multivariate trend observed for other nutrients. Dim2 records further variability (16%) and is mainly determined by K and P (their loadings are −0.664 and 0.623, respectively). Cu and Si also contribute moderately to this component, suggesting that there was greater treatment-specific variation in these elements in 2024. The opposite directions of K (negative) and P (positive) along Dim2 suggest different responses of cations and phosphate accumulation to foliar treatments. Overall, the 2024 PCA structure highlights Fe, Mg, S, and Na as the dominant determinants of treatment-related differences, while Dim2 reveals secondary but still significant contrasts determined by K, P, and Cu. Compared to previous years, the negative loading of Si on Dim1 indicates a clear shift in the multi-element pattern affecting silicon, which likely reflects changes in the 2024 uptake dynamics or interactions between treatments.

**Figure 8:**
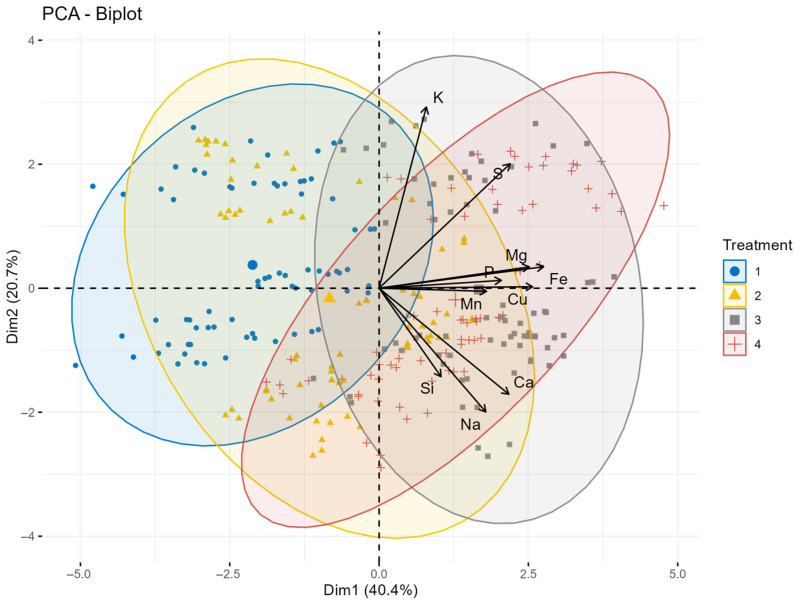
The principal component analysis biplot illustrating the relationship of treatments on macro- and microelement content of oat grain averaged over the years 2022–2024. The first principal component (Dim1) explains 49.0% of the variance, while the second principal component (Dim2) contributes 16.0%, making a total variability of 65.0%. Treatments: 1 = control, 2 = Si, 3 = S, 4 = Si + S foliar treatments.

The results of PCA performed on the combined dataset for 2022–2024 are shown in Figure 8. The results show a stable, year-independent, multi-component pattern in relation to foliage treatments. The first principal component (Dim1), which explains 40.4% of the total variance, is primarily determined by Fe, Cu, Mg, and S (load 0.77–0.85), but Ca also contributes significantly. These elements therefore represent the dominant axis along which the effect of the treatment was consistently manifested in all three years. Na and Si show a moderately negative or nearly neutral correlation with PC1, indicating a weaker or more variable response.

The second principal component (Dim2), which accounts for 20.7% of the variation, is strongly influenced by K and S (with loadings of 0.901 and 0.619, respectively), suggesting that potassium dynamics—and to a lesser extent sulfur—were responsible for the secondary differentiation between treatments. Other nutrients, including Fe, Mg, and P, contribute only marginally to Dim2, reflecting their more stability over time.

Overall, the two principal components together explain 61.1% of the total variability, indicating that the effect of treatments on the composition of oat grains follows robust, multi-year trends.

## 3. Discussion

Numerous studies confirm that oats are rich in essential elements, possess high nutritional value, and provide associated health benefits [29]. The most important finding of this study is that the effects of silicon (Si) and sulfur (S) foliar fertilization on the mineral composition of spring oat grain were strongly dependent on environmental conditions. While combined Si + S treatment proved to be the most effective under severe drought conditions (2022), sole sulfur application was more beneficial under mild (2023) or moderate drought (2024). These findings indicate that physiological role of Si and S differ depending on environmental conditions, particularly drought stress intensity, and that these differences substantially affect the accumulation of macro- and microelements in oat grains. The use of foliar fertilization under variable climatic conditions is therefore essential for optimizing and grain nutrient composition and supporting the long-term sustainable production of spring oats.

Temperature and water availability fundamentally alters plant water relations, ion transport, and metabolic activity, thereby modifying nutrient uptake and allocation. Drought stress is among the most critical factors influencing the elemental composition of cereal grains [30]. The present results clearly demonstrate that environmental conditions were the dominant drivers shaping grain elemental composition, overriding the effects of foliar fertilization alone. The Pálfai Drought index (PaDI) showed strong positive correlations with mobile elements such as K and S, while negative correlations were observed for Ca, Na, Cu, and P. This pattern suggests that increasing drought severity induces a systematic reorganization of ion uptake and allocation, reinforcing the primacy of environmental stress over fertilization effects. This observation is consistent with earlier reports indicating that precipitation and temperature regimes exert a stronger influence on cereal grain mineral profiles than individual agronomic inputs. Studies on spring barley have shown that differing precipitation regimes can significantly affect grain quality, with lower precipitation associated with increased concentrations of certain minerals such as Cu and Na, strongly suggesting that rainfall directly influences cereal grain mineral composition [31].

Rising temperatures during the growing season, combined with extreme heat, prolonged drought, and intense rainfall events, are expected to become increasingly common due to climate change. These shifts in climatic patterns may alter agricultural production by affecting germination, vegetative growth, and final grain quality, underscoring the need to develop adaptive agronomic strategies [32]. Sulfur and silicon applications have been widely reported to influence nutrient uptake and elemental composition in cereals, particularly under environmental stress. However, the present study extends existing knowledge by demonstrating that drought severity also determines whether Si and S act synergistically, independently, or even antagonistically with respect to nutrient accumulation.

Sulfur, as an essential macronutrient, is a key component of amino acids, proteins, and enzymes, and its availability strongly affects nitrogen assimilation and overall nutrient use efficiency. Adequate sulfur supply enhances the uptake and internal utilization of N, P, and K, while also influencing the accumulation of several macro- and microelements through enhanced metabolic activity and redox regulation [33,34]. These processes require active metabolism and sufficient water availability, conditions that are more likely to persist under mild or moderate drought. In our experiment, foliar sulfur application resulted in a statistically significant increase in K concentration, while no significant differences were observed in Na content under severe drought stress. This response suggests that sulfur primarily enhances internal nutrient utilization and metabolic efficiency rather than acting as a direct stress-protective agent. Similar effects of sulfur foliar application have been reported for lupins [35] as well as increased Mn and Cu contents in oat grains [36,37]. In the present study, Mg and Ca contents also increased significantly following sulfur foliar fertilization. These results partly contradict those of Brodowska and Kaczor [38], who also observed increased Mg but decreased Ca concentrations in spring wheat.

Although silicon is not considered an essential element, it plays a beneficial role in regulating the uptake, translocation, and accumulation of macro- and microelements. Several studies have shown that Si application can modify ion availability, enhance root function, and regulate ion transporter activity, leading to altered concentrations of elements such as Ca, Mg, P, Fe, and Mn in plant tissues, while Cu and Zn responses depend on species and environmental conditions. Silicon can reduce the binding of phosphorus (P) to iron and aluminum hydroxides, thereby increasing P availability [39,40]. These effects are often more pronounced under drought stress, where Si improves plant water status and ionic homeostasis. Previous studies have reported that silicon application under optimal production conditions, including irrigation, had no significant effect on grain P, Mg, and Ca content [41]. In contrast, under mildly dry conditions, our study showed statistically significant increases in Ca, P, and Mg content following Si foliar application. Studies on wheat genotypes treated with Si foliar application [42] have shown that under severe drought stress, K content decreases while P concentration increases significantly, which is consistent with our results. In contrast, our results showed that under severe drought stress, Ca content decreased in response to Si foliar fertilization. In addition, oats have a higher Na and Ca content than other cereals such as barley, rye, triticale, sorghum, and millet, making them a valuable component of the human diet [43].

A previous study [44] reported that the Na content of sugar beet roots decreased by up to 54.4% after Si foliar treatment compared to the control treatment. Similar results were observed in our study, where foliar treatment with Si led to a significant decrease in Na content, especially in the fourth variety (‘Panni’), where a 43.3% decrease was observed during severe drought. In contrast, the change in Na content was smaller in other varieties, showing a slight decrease or increase between varieties. In addition, Si treatment affected the uptake of K, S, Mg, Na, Si, Fe, Mn, and Cu nutrients in sunflower varieties during drought [23]. In line with these results, our experiment also showed a significant increase in Mg, Cu, and Si content. However, in the case of K, S, Ca, Fe, and Mn, Si foliar treatment had a negative effect, while in the case of Na, no significant difference was observed during severe drought.

A particularly important and unexpected finding of this study was that under severe drought, sole Si application increased grain Si concentration while exerting neutral or negative effects on most other macro- and microelements. This apparent contradiction of some literature reports warrants careful interpretation, suggesting that there is a difference between silicon accumulation and general nutrient uptake. Silicon is involved in stress defense functions and does not necessarily improve the uptake of other nutrients under severe drought conditions [44]. Silicon contributes to drought tolerance by enhancing cell wall rigidity, reducing cuticular transpiration, and maintaining tissue integrity. However, these protective mechanisms may simultaneously reduce transpirational flow and mass transport of other nutrients, thereby limiting their uptake and translocation.

The decoupling observed between increased Si accumulation and reduced uptake of other elements suggests that under extreme water limitation, silicon functions primarily as a stress-mitigating element rather than a facilitator of general nutrient acquisition. Antagonistic interactions between Si and cations such as Ca^2+^, Mg^2+^, and Fe^2+^, as well as reduced root uptake capacity, may further contribute to the observed decreases in mineral concentrations. These findings highlight that Si application under severe drought does not universally enhance mineral nutrition and may even suppress it in the absence of complementary metabolic support. The Pearson correlation results provide further insight into the contrasting behavior of silicon under drought stress. Grain Si content showed a significant negative correlation with the Pálfai Drought Index (PaDI), indicating enhanced Si accumulation under more severe drought conditions, while its correlations with other mineral nutrients were generally weak or element-specific. In particular, the negative association between Si and K suggests potential antagonistic interactions or shifts in ion transport priorities under water-limited conditions.

Based on our results, the best treatment under severe drought conditions was the Si + S combination, which is consistent with reports that silicon application alters the dynamics of plant nutrient uptake by enhancing iron and potassium uptake while simultaneously helping to inhibit excessive sodium accumulation [45]. Combined Si and S applications may exert synergistic effects, as Si-mediated stress alleviation supports nutrient transport and homeostasis, while sulfur enhances biochemical processes related to protein synthesis and antioxidant defense. Together, these mechanisms can lead to pronounced changes in grain elemental composition, particularly under drought conditions, as observed in cereal crops including oats.

In our current study, we determined the concentrations of K, P, S, Mg, Ca, Na, Fe, Mn, Cu, and Si in oat grains of eight genotypes, and the results were largely consistent with those reported in previous studies [46,47]. Statistically significant differences in macro- and microelement concentrations between varieties reflect the genetic diversity of oats. These results confirm previous studies that the quality of oat grains is strongly influenced by both genetic variation and environmental conditions [48,49,50]. Consequently, variety selection and seasonal factors are critical for optimizing the mineral content of oats. Özcan et al. [5] reported Cu concentrations ranging from 2.67 to 15.86 mg kg^−1^ for different oat varieties. Similar values were recorded in our study, where the Cu content ranged from 4.1 to 15.9 mg kg^−1^, depending on the variety and treatment.

According to Loskutov [1], the Fe content of the analyzed oats ranges between 19 and 37 mg kg^−1^, while the data in this study show higher concentrations, ranging between 52.6 and 59.9 mg kg^−1^ in different years. Similarly, the Mn content of the samples examined by Loskutov [1] was 3.5–9.9 mg kg^−1^, while the results of the present study showed higher contents, ranging from 50.5 to 55.6 mg kg^−1^, under different climatic conditions. Biel et al. [2] reported Fe and Cu contents of 41 mg kg^−1^ and 4 mg kg^−1^, respectively, in hulled oats, which is very similar to the results of our study, where we recorded averages of 44.2 mg kg^−1^ Fe and 4.6 mg kg^−1^ Cu under moderately dry climatic conditions. Although Cu and Mn are essential micronutrients, excessive accumulation may be harmful for humans’ health; in the present study they fall within the ranges reported for cereal grains.

These results underline the importance of genotype-specific nutrient management strategies. Varieties with higher Si accumulation capacity or more efficient Na exclusion may benefit more from Si-based treatments, particularly under drought conditions. The interaction between genotype, fertilization strategy, and environmental stress highlights the complexity of optimizing grain nutritional quality under changing climatic conditions.

Several findings of the present study differ from earlier reports, particularly regarding the effects of Si on macroelement accumulation. These discrepancies likely arise from differences in drought severity, timing of stress exposure, application method (foliar versus soil), soil nutrient status, and genotypic background. Many studies reporting positive effects of Si on nutrient uptake were conducted under well-watered or moderately stressed conditions, where transpirational flow and root activity were less constrained. Our results emphasize that the effects of Si and S are highly context-dependent rather than universally positive or negative. Understanding these context-specific responses is essential for interpreting inconsistencies in the literature and for developing effective fertilization strategies under variable environmental conditions.

The effect of Si is not uniform across all plant species and depends on the plant’s ability to accumulate Si, the Si content of the soil, and the presence of other nutrients [51]. The results of our study show that the nutritional quality of oats is determined by the interaction of silicon and sulfur foliar fertilization, genetic variations, and environmental conditions. In addition, annual climatic changes, such as changes in temperature and water availability, significantly influenced the accumulation of nutrients in oat varieties. Foliar fertilization and silicon had different effects on elemental composition, which was highly dependent on crop years, highlighting the integrated approach of agronomic management. These interactions indicate that genetic variation and foliar fertilization play an important role in improving the nutritional quality of oats under changing environmental conditions.

A limitation of this study is the lack of direct physiological measurements such as transpiration rate, antioxidant enzyme activity, or root hydraulic conductance, which would allow a more precise mechanistic interpretation of nutrient responses. In addition, the number of growing seasons limits the generalization of the findings across broader climatic gradients. Despite these limitations, this study provides novel insights into the interaction between drought severity, foliar Si and S fertilization, and genotypic variation in spring oats. The documentation of negative or neutral effects of sole Si application under severe drought, alongside the demonstrated benefits of combined Si + S treatment, represents a significant contribution to the understanding of stress-adaptive nutrient management. From a practical perspective, the results suggest that foliar fertilization strategies should be tailored to drought intensity. Under mild or moderate drought, sulfur application alone may be sufficient to enhance grain mineral composition, whereas under severe drought, combined Si and S application appears more effective. These findings support the development of adaptive, environment-specific fertilization strategies to improve the nutritional quality and resilience of oat production under increasingly variable climatic conditions.

## 4. Materials and Methods

### 4.1. Soil Characteristics of the Experimental Site

The experiment was carried out in the spring–summer of 2022, 2023, and 2024 at the University of Debrecen Experimental Garden in Hungary, at the coordinates 47°55′19″ N–21°59′87″ E. In early March 2022, prior sowing the oat experiment, soil samples were collected from a depth of 200 cm. Comprehensive soil analyses were performed for ten soil layers at 20 cm depth intervals. The soil analysis was conducted at the Agricultural Laboratory Centre of the University of Debrecen. This laboratory is accredited by the National Accreditation Board of Hungary.

The humus content of the upper soil layer (0–60 cm depth) is exceptionally good (2.63–2.89%). Soil pH in the topsoil is almost neutral (pH_KCl_ = 6.93), while in subsoil layers it is slightly alkaline. The Arany-type plasticity index (K_A_ = 45–52) suggests medium-textured loam soil. Calcium carbonate content in the surface layer is low (0.52%), but increasing with the depth, indicating the presence of a calcic horizon at 50–100 cm depth. The topsoil is exceptionally rich in phosphorus (P_2_O_5_ (AL) = 1538 mg kg^−1^), potassium (K_2_O (AL) = 638 mg kg^−1^), magnesium (Mg (KCl) = 552 mg kg^−1^) (Table 8). Based on these characteristics, according to the WRB 2022 soil classification system [52], the soil at the experimental site is classified as Haplic Chernozem with calcic properties (Calcic Chernozem). The deep, well-developed soil profile and favorable physical properties provide suitable conditions for root development and nutrient uptake in oat. Although the majority of the roots of spring oat are generally concentrated in the upper 0–60 cm layer, the maximum rooting depth may reach 1.0–1.2 m under favorable conditions, which is particularly relevant under drought conditions.

### 4.2. Climatic Conditions and the Calculation of the Pálfai Drought Index (PaDi)

The daily temperature and precipitation data were measured at the University of Debrecen’s Experimental Garden, the site of the experiment. Meteorological data were provided by the Agrometeorological Observatory of the Centre for Precision Farming R&D Services, FAFSEM, University of Debrecen.

During the spring oat cultivation period (March to July), average temperatures showed notable variation across the three experimental years, directly influencing plant growth and development. In 2022, temperatures were lower during germination and early growth (March: 5.2 °C and April: 9.7 °C), whereas in 2023 and 2024 they were higher (March: 7.5 °C and 9.9 °C; April: 10 °C and 14 °C), creating more favorable conditions for early sowing, germination, and initial development. In the subsequent months, temperatures followed an increasing trend, with 2024 recording the highest values in May (18 °C) and July (25.2 °C), potentially contributing to greater temperature instability compared with the other years.

**Figure 9:**
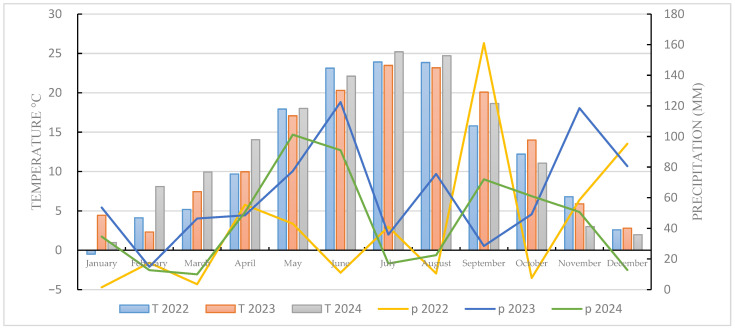
Monthly average temperature and monthly precipitation sum in the study area over a three-year period (Debrecen, 2022–2024).

Analysis of precipitation during the spring oat growing period (sowing to harvest) also revealed substantial yearly differences that may have affected grain quality. The year 2023 was characterized by more favorable climatic conditions, with evenly distributed precipitation—46.4 mm in March, 48.5 in April, 77.4 mm in May, 122.5 mm in June, and 35.9 mm in July—providing sufficient moisture throughout all developmental stages. In contrast, 2022 experienced minimal precipitation in March (3.5 mm) and a significant deficit in June (11 mm). The year 2024 showed considerable variability, with March and June receiving 78.4%, 25.7% less precipitation than 2023, and July receiving 58.5% less precipitation than 2022, while precipitation in May was 57.5% and 23.5% higher than in 2022 and 2023, respectively (Figure 9).

The Pálfai Drought Index was used to characterize the severity of water scarcity in the years studied. To calculate the modified Pálfai Drought Index [28], the base-value is first calculated:

$${PaDI}_{0}=\frac{\frac{\left[\sum_{i=apr}^{aug}T_{i}\right]}{5}*100}{c+\sum_{i=oct}^{sept}\left(P_{i}*w_{i}\right)}$$

${PaDI}_{0}$ = base-value of drought index (°C 100 mm^−1^);
$T_{i}$ = monthly mean temperature from April to August (°C);
$P_{i}$ = monthly sum of precipitation from October to September (mm);
$w_{i}$ = weighting factor (Table 9);
c = constant value (10 mm).

**Table 9:** Weighting factor (w_i_) values for calculating the Pálfai Drought Index [28].

Month	w_i_
October	0.1
November–December	0.4
January–April	0.5
May	0.8
June	1.2
July	1.6
August	0.9
September	0.1

Calculation of drought index correction factors (k_1,_ k_2_, k_3_):

$k_{1}$ = temperature correction factor:

$$k_{1}=\frac{\left(T_{jun}+T_{jul}+T_{aug}\right)/3}{\left({\bar{T}}_{jun}+{\bar{T}}_{jul}+{\bar{T}}_{aug}\right)/3}$$

$T_{jun}+T_{jul}+T_{aug}$ = annual mean temperature for June–August (°C);

${\bar{T}}_{jun},{\bar{T}}_{jul},{\bar{T}}_{aug}$ = 30-year mean temperature for June–August for period 1991–2020 (°C);

$k_{2}$ = precipitation correction factor:

$$k_{2}=\sqrt[{4}]{\frac{2*{\bar{P}}_{summer}^{min}}{MIN\left(P_{jun},P_{jul},P_{aug}\right)+{\bar{P}}_{summer}^{min}}}$$

${\bar{P}}_{summer}^{min}$ = the lowest value from 30-year precipitation sum of three summer months (June, July, August) (mm);

${MIN(P}_{jun},\;P_{jul},\;P_{aug})$ = the lowest value from annual precipitation sum of three summer months (June, July, August) (mm);

$k_{3}$ = correction factor, which characterizes the precipitation circumstances of the previous period:

$$k_{3}=\sqrt[{n}]{\frac{\bar{P}}{{\bar{P}}_{36m}}}$$

*n* = reduction factor; value is 3.0 on the plain area, 5.0 on hilly or higher territories;
$\bar{P}$ = average multi-annual precipitation sum for period October-September (mm);
${\bar{P}}_{36m}$ = average precipitation sum for period October-September for previous three years (mm):
  $${\bar{P}}_{36m}=\sum_{i=\left(year-3\right)Oct}^{\left(year\right)Sept}P_{i}/36$$
$P_{i}$ = monthly precipitation sum (mm).

Calculation of the Pálfai Drought Index (PaDI) (°C 100 mm^−1^):

$$PaDI={PaDI}_{0}*k_{1}*k_{2}*k_{3}$$

### 4.3. Experimental Design and Treatment

The spring oat small plot (3 × 4 = 12 m^2^) experiment was conducted as a randomized complete block design (RCBD). Eight varieties were sown in 3 m wide strips placed side by side. The four treatments were arranged in four blocks, one behind the other, with three repetitions in each block. In total, the experiment consisted of 96 plots (8 varieties × 4 treatments × 3 replications). To ensure crop rotation, the location of the experiment within the Experimental Garden was changed from year to year. The forecrops were winter wheat (2022); bean (2023); soybean (2024).

The experiment was sown on 18 March 2022, 7 March 2023, and 6 March 2024, applying a seed rate of 550 seeds/m^−2^, at the depth of 5 cm. A uniform NPK fertilization was applied in the experiment (2022: N_5_P_10_K_30_ kg ha^−1^ in October 2021 and N_55_ kg ha^−1^ in March 2022; 2023: N_55_ kg ha^−1^ in March 2023; 2024: -). In 2023, lodging was considerable in the experiment due to the good nutrient supply of the soil, the excellent nutrient use efficiency of oat, the favorable preceding crop (legume), and relatively adequate water supply. Consequently, no supplementary NPK fertilization was applied in 2024.

Uniform plant protection was applied in the experiment:

• Seed dressing: tebuconazole;
• Pest control: lambda-cihalotrin 0.15 L ha^−1^ against cereal leaf beetle (*Oulema melanopus*) at end of tillering (BBCH29), and at end of flowering (BBCH69).

The eight tested spring oat (*Avena sativa* L) varieties (i.e., ‘GK Kormorán’, ‘GK Pillangó’, ‘Lota’, ‘Panni’, ‘Mv Kengyel’, ‘Mv Ménes’, ‘Mv Pehely’, ‘Mv Szellő’) are promising Hungarian genotypes.

We applied four treatments:

1. Control, without fertilization;
2. Silicon fertilization (Si) 0.5 L ha^−1^;
3. Sulphur fertilization (S) 5.0 L ha^−1^;
4. Silicon + Sulphur fertilization (Si + S) 0.5 + 5.0 L ha^−1^.

The applied foliar fertilizers using a spraying volume of 300 L ha^−1^ were:

• S: Jello Fluid (Kwizda Agro, Budapest, Hungary): liquid foliar fertilizer with high content S (lignosulfonate formulation) 1000 g L^−1^ SO_3_, 30 g L^−1^ N, 30 g L^−1^ MgO, 27 g L^−1^ B, 0.003 g L^−1^ Mo;
• Si: Optysil (Intermag, Olkusz, Poland) with high content of Si: (200 g SiO_2_ L^−1^).

The foliar fertilizers were applied to oat leaves at three developmental stages, namely three leaves unfolded (BBCH13), flag leaf stage (BBCH39), and early milk stage (BBCH73), using a nonionic adjuvant containing 60% dioctyl sulfosuccinate sodium salt at a concentration of 0.025%.

The oats were harvested on 11th of July for 2022 and 2024, and 24th July for 2023 by using a Wintersteiger 125 plot combine with 125 cm cutting width. Grain samples were taken from each plot for further grain quality analysis.

### 4.4. Analytical Method of Grain Element Content

The oat grain samples first were ground and afterward they underwent preparation via wet acid digestion, which consisted of two consecutive stages: pre-digestion and digestion. A precisely 1 g portion of the oat grains was weighed and then transferred into a digestion tube, to which 10 mL of nitric acid (HNO_3_, 69% *v*/*v*) (VWR International Ltd., Radnor, PA, USA) was added. The early pre-digestion phase required a 12 h reaction period, followed by heating the samples at 60 °C for 30 min using a LABOR MIM OE-718/A block digestion system (LABOR-MIM, Budapest, Hungary). After allowing the samples to cool briefly, 3 cm^3^ of hydrogen peroxide (H_2_O_2_, 30% *v*/*v*) (VWR International Ltd., Radnor, PA, USA) was added to each sample.

In the digestion phase, the temperature was elevated to 120 °C for 90 min to ensure complete mineralization of the organic matrix. Following digestion and cooling, the final volume of the digested solution was adjusted to 50 cm^3^ using ultrapure water (Milli-Q two-level water purification system, Millipore S.A.S, Molsheim, France). The digested solution was subsequently filtered using Filtrak 388 filter paper before elemental analysis via an Inductively Coupled Plasma Optical Emission Spectrometer (iCAP 7400 ICP-OES, Thermo Fisher Scientific Inc., Waltham, MA, USA). The quantification of macroelement concentration (P, Mg, N, K) and microelement concentration (S, Si, Cu, Mn, Fe) was conducted using a PerkinElmer Optima 3300 DV ICP-OES (PerkinElmer Inc. Waltham, MA, USA). This instrument simultaneously and with high precision determined multiple element content. Grain element content was analyzed according to the standardized Hungarian methods (MSZ EN 15510:2017 standard) [53], which clearly define sample preparation techniques, digestion, and measurement conditions for the ICP-OES, and with an allowed analytical deviation of ±10%, to ensure acceptable precision and repeatability.

### 4.5. Data Analysis

All processed data were subjected to detailed statistical analysis using the advanced Gen-Stat program, version 18 (VSN International, Rothamsted, UK). Two-factor analysis of variance (ANOVA) was used to analyze the effects of the treatments and to compare the mean values. The variety × treatment interaction was also analyzed. During the variance analysis, we examined the prerequisites (normality, homogeneity). Subsequently, a three-factor analysis of variance was conducted to evaluate the Treatment × Variety × Year interaction. We compared the mean values of all components of the oat grain between varieties and treatments using the least significant difference (LSD) at a 5% probability level. We analyzed each year separately. The linear correlations between the parameters were discovered using Pearson correlation analysis (2-tailed). To evaluate treatment-induced differences in the macro- and microelement composition of oat grain and to identify the main factors driving variability among treatments, Principal Component Analysis (PCA) was applied with R statistics v. 4.5.0 [54]. PCA is a multivariate statistical method that reduces the dimensionality of complex datasets by transforming correlated variables into a set of orthogonal principal components that capture the majority of the original variance. This approach facilitates the visualization of treatment-related clustering and highlights the elements contributing most strongly to differences in grain elemental composition.

## 5. Conclusions

The application of sulfur and silicon, both individually and in combination, influenced the macro- and microelement composition of spring oat grain; however, the magnitude and direction of these effects were strongly dependent on drought severity and genotype. Sulfur-containing treatments (sole S and combined Si + S application) consistently resulted in statistically significant increases in grain nutrient concentrations, indicating that sulfur availability was the most robust and reliable driver of improved mineral composition. Sole sulfur application proved to be the most effective treatment, markedly increasing the concentrations of key macronutrients (K, P, S, Ca) and micronutrients (Na, Fe, Mn, Cu), with the exception of Na under severe drought stress. In contrast, foliar silicon application alone showed limited or, under severe drought conditions, neutral to negative effects on most nutrients, despite increased silicon accumulation in grain. This finding suggests that under extreme water limitation, silicon primarily contributes to stress mitigation rather than enhanced nutrient uptake. This context-dependent response of silicon represents a key outcome of the study and highlights that its agronomic effectiveness cannot be generalized across climatic conditions. Genotypic differences further modulated nutrient accumulation patterns, confirming that genetic background interacts with environmental stress and foliar treatments in shaping grain elemental profiles of spring oat grain. Although the three-year dataset under contrasting climatic conditions strengthens the reliability of these findings, the site-specific nature of the experiment, soil fertility status, and climatic variability limit broader generalization. Therefore, further multi-location and multi-year studies involving diverse environment and soil types and a wider range of genotypes are required to refine environment-specific foliar fertilization strategies aimed at improving the nutritional quality and resilience of spring oat production under increasingly variable climatic conditions.

## Figures and Tables

**Table 1:** The calculated Pálfai Drought Index during three growing seasons in the experimental site in Debrecen (2022–2024).

Year	P_36 month_ mm	Monthly Average P for Previous 36 Month mm	Sum of Precipitation April–August mm	${MIN(P}_{jun},P_{jul},P_{aug})$ mm	PaDI_0_ °C 100 mm^−1^	PaDI °C 100 mm^−1^
2022	1519.7	42.2	160.9	10.5	8.75	11.6
2023	1567.3	43.5	359.8	35.9	3.76	4.3
2024	1761.7	48.9	282.2	17.0	5.19	6.5

Note: P_36month_ = precipitation sum for period October–September for previous three years; ${MIN(P}_{jun},\;P_{jul},\;P_{aug})$ = the lowest value from annual precipitation sum of three summer months (June, July, August).

**Table 2:** Drought categories according to PaDI [28].

PaDI °C 100 mm^−1^	Classification
<4	droughtless year
4–6	mild drought
6–8	moderate drought
8–10	medium drought
10–15	serious drought
15–30	very serious drought
>30	extreme drought

**Table 3:** A grand mean for the impact of changes in the genetic aspects of selected varieties on the reflection of macroelements and microelements measured in spring oat grain, in average of years and treatments (Debrecen, 2022–2024).

Variety	Macroelements (mg kg^−1^)					Microelements (mg kg^−1^)				
	K	P	S	Mg	Ca	Na	Si	Fe	Mn	Cu
GK Kormorán	4976	4885	1930	1618	1064	121	69.7	61.4	59.3	10.4
GK Pillangó	4903	5156	1833	1546	1007	128	63.7	55.5	53.1	8.7
Lota	4773	5436	1920	1607	1097	128	68.2	58.5	54.5	10
Panni	4995	5610	1918	1566	1020	121	81.5	54.7	52.7	10.5
Mv Kengyel	5022	5098	1789	1550	1015	116	65.2	53	53.3	9.1
Mv Ménes	4931	4925	1804	1593	1127	142	68.5	53.4	54	10.7
Mv Pehely	5425	5710	2053	1662	994	141	55	57	51.8	11.1
Mv Szellő	4912	5493	1915	1544	1022	136	72.2	56	51.7	10.7
LSD_0.05_	43	21.7	18	17.5	15.8	4.8	1.99	0.86	0.53	0.18

Note: LSD_0.05_: Least Significant Difference at *p* = 5%.

**Table 4:** A grand mean for the impact of nutritional supply on the reflection of macroelements and microelements measured in spring oat grain in average of years and varieties (Debrecen, 2022–2024).

Treatment	Macroelements (mg kg^−1^)					Microelements (mg kg^−1^)				
	K	P	S	Mg	Ca	Na	Si	Fe	Mn	Cu
Control	4780	4859	1768	1493	924	107	47.3	50.5	48.4	7.3
Si	4841	5075	1802	1559	958	124	80	53.7	50.8	9.2
S	5250	5623	2032	1640	1179	146	61.5	61.5	58.9	12.3
Si + S	5098	5600	1979	1651	1113	139	83.3	59.1	57.1	11.8
LSD_0.05_	32.4	20.4	14.8	11.6	11.4	2.7	1.26	0.7	0.47	0.13

Note: LSD_0.05_: Least Significant Difference at *p* = 5%.

**Table 5:** Effect of environmental–season conditions on the macro- and microelement concentration in the grain of spring oats in average of treatments and varieties (Debrecen, 2022–2024).

Year	Macroelements (mg kg^−1^)					Microelements (mg kg^−1^)				
	K	P	S	Mg	Ca	Na	Si	Fe	Mn	Cu
2022	7153	4913	2077	1608	930	110	55.4	56.1	55.2	9.3
2023	4869	6501	1862	1580	1110	137	76.1	59.9	50.5	13.6
2024	2954	4453	1746	1569	1090	140	72.6	52.6	55.6	7.5
LSD_0.05_	25.7	15.3	11.6	9.8	8.5	2.3	0.99	0.51	0.33	0.13

Note: LSD_0.05_: Least Significant Difference at *p* = 5%.

**Table 6:** Treatment × Variety × Year interactions (Debrecen, 2022–2024).

Element	df	F	* p *	Partial η ^2^
Si	42	33.66	0.000	0.88
P	42	29.57	0.000	0.87
Cu	42	28.70	0.000	0.86
Ca	42	26.38	0.000	0.85
K	42	17.40	0.000	0.79
S	42	8.25	0.000	0.64
Mn	42	7.29	0.000	0.61
Na	42	5.97	0.000	0.57
Mg	42	5.65	0.000	0.55
Fe	42	3.91	0.000	0.46

Note: df: degree of freedom; F: F-value; *p*: significance; partial η^2^: partial eta square.

**Table 7:** Pearson correlation coefficient values among treatments, sum of precipitation during the growing season, PaDI values, and element content in spring oat whole grains (Debrecen, 2022–2024).

	Treatment	P_03–07_	PaDI	Ca	Cu	Fe	K	Mg	Mn	Na	P	S	Si
Treatment	1	0.000	0.000	0.497 **	0.500 **	0.542 **	0.086	0.578 **	0.596 **	0.483 **	0.288 **	0.437 **	0.368 **
P_03–07_	0.000	1	−0.999 **	0.442 **	0.380 **	0.152 *	−0.663 **	−0.121 *	−0.257 **	0.440 **	0.497 **	−0.475 **	0.328 **
PaDI	0.000	−0.999 **	1	−0.447 **	−0.352 **	−0.132 *	0.696 **	0.125 *	0.245 **	−0.450 **	−0.465 **	0.494 **	−0.330 **
Ca	0.497 **	0.442 **	−0.447 **	1	0.411 **	0.464 **	−0.281 **	0.463 **	0.415 **	0.684 **	0.363 **	0.166 **	0.234 **
Cu	0.500 **	0.380 **	−0.352 **	0.411 **	1	0.678 **	0.191 **	0.396 **	0.191 **	0.344 **	0.796 **	0.437 **	0.338 **
Fe	0.542 **	0.152 *	−0.132 *	0.464 **	0.678 **	1	0.275 **	0.568 **	0.451 **	0.385 **	0.517 **	0.561 **	0.178 **
K	0.086	−0.663 **	0.696 **	−0.281 **	0.191 **	0.275 **	1	0.268 **	0.070	−0.342 **	0.168 **	0.687 **	−0.266 **
Mg	0.578 **	−0.121 *	0.125 *	0.463 **	0.396 **	0.568 **	0.268 **	1	0.548 **	0.446 **	0.255 **	0.629 **	0.146 *
Mn	0.596 **	−0.257 **	0.245 **	0.415 **	0.191 **	0.451 **	0.070	0.548 **	1	0.241 **	−0.093	0.435 **	0.198 **
Na	0.483 **	0.440 **	−0.450 **	0.684 **	0.344 **	0.385 **	−0.342 **	0.446 **	0.241 **	1	0.214 **	0.022	0.223 **
P	0.288 **	0.497 **	−0.465 **	0.363 **	0.796 **	0.517 **	0.168 **	0.255 **	−0.093	0.214 **	1	0.346 **	0.205 **
S	0.437 **	−0.475 **	0.494 **	0.166 **	0.437 **	0.561 **	0.687 **	0.629 **	0.435 **	0.022	0.346 **	1	−0.021
Si	0.368 **	0.328 **	−0.330 **	0.234 **	0.338 **	0.178 **	−0.266 **	0.146 *	0.198 **	0.223 **	0.205 **	−0.021	1

Note: P_03–07_: precipitation sum for period March–July; PaDI: Pálfai Drought Index value *: the correlation is significant at *p* = 5%; **: the correlation is significant at *p* = 1%.

**Table 8:** Soil analysis results in the field of the conducted experiment (2022, Debrecen).

Soil Parameters at the Experimental Station	Soil Profile Layers (cm)									
	0–20	20–40	40–60	60–80	80–100	100–120	120–140	140–160	160–180	180–200
pH (KCl)	6.93	7.46	7.51	7.56	7.55	7.59	7.60	7.73	7.74	7.76
K_A_	45	52	52	53	53	56	54	51	50	49
Soluble Salts (%)	0.05	0.05	0.05	0.05	0.05	0.06	0.06	0.05	0.04	0.04
CaCO_3_%	0.52	1.45	1.97	2.07	1.86	2.48	2.80	2.90	3.00	3.11
Humus (%)	2.89	2.87	2.63	2.44	2.37	2.55	1.22	0.741	0.445	0.407
P_2_O_5_ (AL) (mg kg^−1^)	1538	1149	1020	390	321	954	229	161	105	97.5
K_2_O (AL) (mg kg^−1^)	638	586	366	315	103	421	146	97.8	92.1	87.5
NO_3_^¯^ (KCl) (mg kg^−1^)	82.2	53.4	48.5	36.5	31.6	144	98.8	82.5	66.5	54.6
Na (Al) (mg kg^−1^)	73.7	68.6	66.9	52.1	50.4	107	99.3	91.3	87.7	86.3
Mg (KCl) (mg kg^−1^)	552	463	352	324	269	584	694	664	540	450
S (KCl) (mg kg^−1^)	14.1	7.31	3.69	3.38	2.43	4.60	47.8	39.6	36.4	32.6
Mn (EDTA) (mg kg^−1^)	25.4	25.2	21.7	15.6	13.2	17.6	15.4	10.2	9.36	7.96
Zn (EDTA) (mg kg^−1^)	2.77	2.24	1.84	1.36	1.21	2.19	1.21	1.13	0.851	0.726
Cu (EDTA) (mg kg^−1^)	1.27	1.15	1.03	0.967	0.952	1.09	0.555	0.533	0.345	0.297
Soluble Si (mg kg^−1^)	35.8	28.0	23.0	6.65	5.66	14.8	3.96	3.18	3.74	3.09

Note: KA: Arany-type plasticity; AL: ammonium lactate-soluble; KCl: potassium chloride soluble; EDTA: Ethylenediaminetetraacetic acid.

## Data Availability

The data presented in this study are available on request from the corresponding author.
